# Rare occurrence of inflammatory bowel disease in a cohort of Han Chinese ankylosing spondylitis patients- a single institute study

**DOI:** 10.1038/s41598-017-13573-z

**Published:** 2017-10-13

**Authors:** Chrong-Reen Wang, Chia-Tse Weng, Chung-Ta Lee, Kuo-Yuan Huang, Sheng-Min Hsu, Ming-Fei Liu

**Affiliations:** 1 Department of Internal Medicine, National Cheng Kung University Hospital, Tainan, Taiwan; 2 Pathology, National Cheng Kung University Hospital, Tainan, Taiwan; 3 Orthopaedics, National Cheng Kung University Hospital, Tainan, Taiwan; 4 Opthalmology, National Cheng Kung University Hospital, Tainan, Taiwan

## Abstract

Despite a high prevalence of ankylosing spondylitis (AS) in Han Chinese, the clinical experience remains very limited in the extra-articular presentation of inflammatory bowel disease (IBD). A monocentric retrospective study was performed for the AS-associated IBD manifestation. This study analyzed AS patients fulfilling the 1984 revised New York diagnostic criteria, excluding those who had the onset of IBD before or concurrently with the diagnosis of AS, for their demographic, clinical, laboratory, radiological, pathological and medication data, particularly in the usage of anti-TNF monoclonal antibody. Among 988 AS patients with 19.8% female, 4 (0.4%) had the overt IBD presentation, one female and 3 male aged 28 to 47 years (38.8 ± 4.6), all ulcerative colitis with the characteristic histopathological findings. At the onset of colitis, all had a long-term disease duration of 10 to 25 years (17.5 ± 6.5) and high BASDAI 7.5 to 8.8 (8.2 ± 0.5) with the hip joint involvement. There were recurrent flares of colitis despite the treatment with corticosteroids and messalazopyrin/salazopyrin, and no relapses of IBD were observed for 6.0 ± 1.1 years after the adalimumab (ADA) therapy. In this retrospective cohort, we demonstrate the rarity of AS-associated IBD manifestation in Han Chinese with a beneficent effect from the ADA therapy.

## Introduction

Ankylosing spondylitis (AS), a HLA-B27-related rheumatological disorder predominantly involving axial skeleton and peripheral joints, is commonly encountered in the clinical practice^1^. In addition to the spine and joint involvement, comorbidities like cardiovascular risk and osteoporosis complication contribute to the disease burden, and extra-articular manifestations further raise the difficulty in clinical management^2^. The prevalence of AS is between 0.2 to 0.5% in Han Chinese from Taiwan and China, similar to Caucasian from western countries, and the commonest extra-articular presentation is acute anterior uveitis with around 30% occurrences, identical with the frequencies reported form Europe and North America^3–5^. Nevertheless, the clinical experience in the inflammatory bowel disease (IBD) manifestation remains very limited in Han Chinese, whereas 5 to 10% of AS patients from western countries have such a presentation^2,5^. Notably, the introduction of biologics antagonizing TNF has revolutionized the treatment of IBD not responding to the conventional therapy^6^, and the application of TNF inhibitors in axial spine, peripheral joints and extra-articular manifestations of AS is under active pharmacological development^1^. In southern Taiwan with a Han Chinese-dominant population, there is an increasing trend of biologics usage in miscellaneous rheumatological disorders^7,8^. A retrospective study was performed in a monocentric cohort for the AS-associated IBD manifestation, especially in the usage of adalimumab (ADA), an effective TNF monoclonal antibody (mAb) in controlling the articular activities of AS. In addition, English literature was reviewed for the reported effects by using TNF blockades on the AS-associated IBD from different racial groups.

## Results

**Figure 1:**
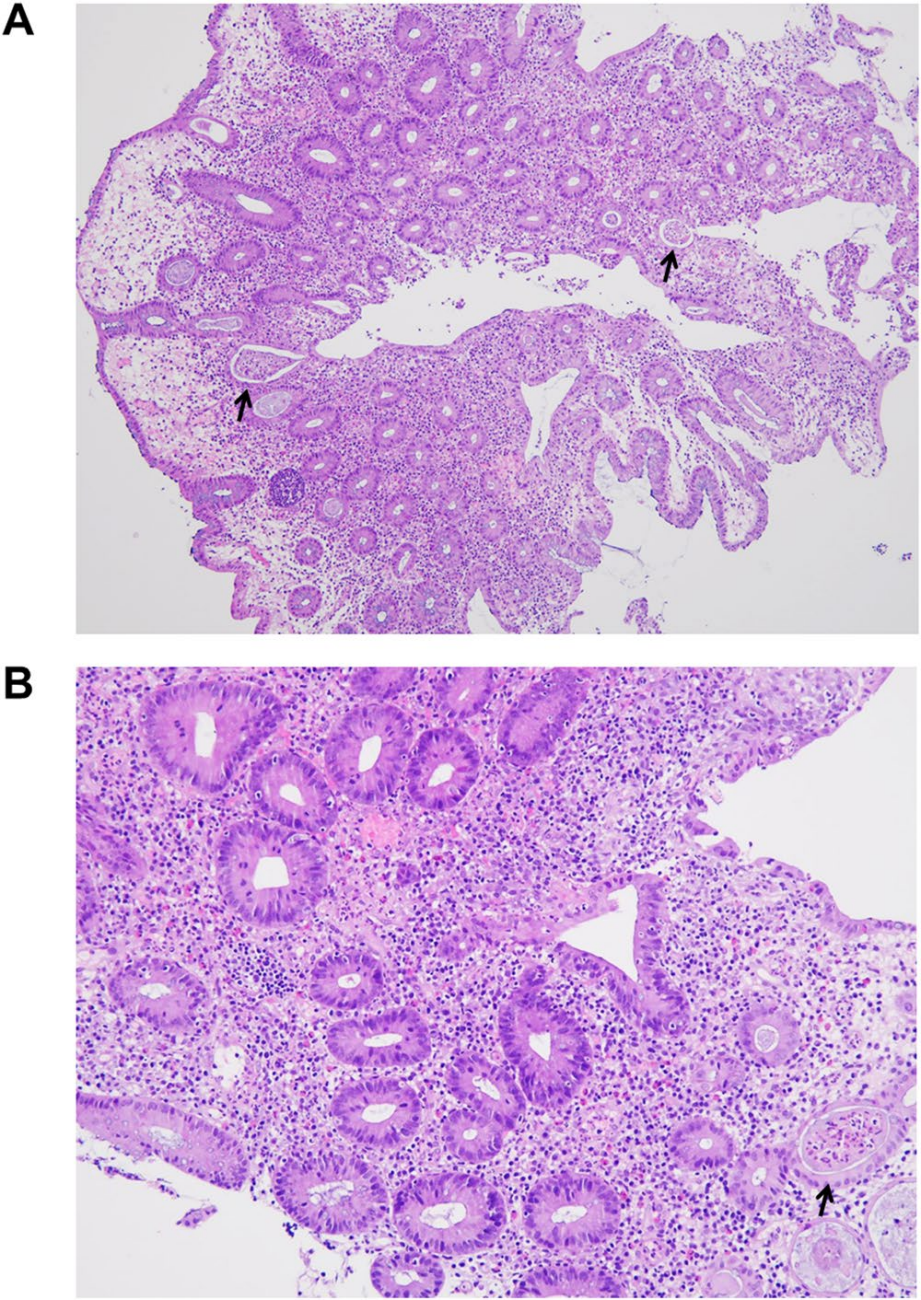
The characteristic histopathological findings of UC on colonic biopsy specimens from case no. 3. (**A**) Mild crypt distortion with the crypt abscess formation (arrows) (hematoxylin and eosin, original magnification, ×40). (**B**) Infiltration of inflammatory cells composed of neutrophils, lymphocytes and plasma cells in the glands and lamina propria with the crypt abscess (arrow) (hematoxylin and eosin, original magnification, ×100).

This monocentric cohort included 988 non-selective consecutive Han Chinese patients, 196 female and 792 male (80.2%) aged 18 to 70 years (32.9 ± 11.8), with a regular follow-up every 1 to 3 months at the Outpatient Department of NCKUH. The IBD manifestation was identified in 4 cases (0.4%), one female and 3 male aged 28 to 47 years (38.8 ± 4.6), all with ulcerative colitis (UC) evaluated by clinical presentations of non-infectious bloody diarrhea, morphological appearances of colon ulcers, and characteristic histopathological findings from intestine biopsy to establish their final diagnosis (Fig. 1)^9^. In Table 1, there were demographic, clinical, laboratory, radiological data, medication profiles, clinical course and final outcome in these patients. At the onset of colitis, there were a long-term disease duration from 10 to 25 years (17.5 ± 6.5), high Bath Ankylosing Spondylitis Disease Activity Index (BASDAI) 7.5 to 8.8 (8.2 ± 0.5), and elevated levels of ESR (35 to 80, 55.0 ± 18.7 mm/hr) and CRP (18.8 to 60.2, 34.4 ± 18.0 mg/L). All had the HLA-B27 genetic marker. In addition to the SI joint and spine, all patients had the hip joint involvement, leading to total hip arthroplasty^10^. For the prescribed medications, nonsteroidal anti-inflammatory drugs (NSAIDs) were replaced with celecoxib, a cyclooxygenase inhibitor not known to exacerbate colitis, after the development of UC, and disease modified anti-rheumatic drugs (DMARDs) usages included methotrexate in 2 and salazopyrin in 4 cases. The clinical manifestations of IBD included fever in 2, and bloody diarrhea with anemia in 4 patients. These cases received high dosages of corticosteroids (1~2 mg/kg/day prednisolone equivalent doses) at the onset of colitis episode. Despites the maintenance usage of corticosteroids and messalazopyrin/salazopyrin, all patients had the relapses of IBD. Two cases expired 4 and 10 years later due to the infection events. ADA was prescribed in case no. 3 for 6.7 years with 40 mg subcutaneous injection every 2 weeks for 4 years, every 3 weeks for 1 year and every 4 weeks for 1.7 years, and in case no. 4 for 5.2 years with 40 mg injection every 2 weeks for 2 years and every 4 weeks for 3.2 years. There was a decrease in BASDAI from 8.8 to 2.8 in no. 3 and 8.1 to 2.6 in no. 4, and no more relapses of UC in both cases for 6.0 ± 1.1 years evaluated by the clinical manifestations and laboratory examinations. Another 64 AS patients, 54 male and 10 female aged from 18 to 70 years (49.9 ± 14.4), had a decrease in BASDAI from 7.7 ± 0.8 to 2.4 ± 1.1 after the ADA therapy. Acute anterior uveitis was identified in 6 cases before receiving this biologics, and there were no recurrences after the therapy for 1.6 ± 1.2 years, consistent with the recently reported effect of ADA on such a manifestation^11^. Furthermore, no ADA-related adverse effects were observed in this study. In addition, etanercept (ETA) injection was prescribed in 24 AS patients, 16 male and 8 female aged from 20 to 68 years (42.1 ± 14.3) during this study period.

**Table 1:** Demographic, clinical, laboratory data, medication profiles, clinical course and final outcome in Han Chinese with the AS-associated UC manifestation.

No.	Age Sex	DP (yr)	Involved joints	Medication before the onset of IBD*	BASDAI/ESR/CRP^†^ at the onset of IBD	IBD clinical/laboratory manifestation	IBD medications	Clinical course	Final outcome
1	28 M	10	SI, spine, hip, shoulder, knee	MTX, NSAIDs, SAZ	8.3/55/31.5	Bloody diarrhea/anemia	Corticosteroids/mSAZ with relapse	Recurrent IBD	Death at 38 y/o due to infection
2	46 M	20	SI, spine, hip	NSAIDs, SAZ	7.5/50/26.9	Bloody diarrhea, fever/ anemia	Corticosteroids/mSAZ with relapse	Recurrent IBD	Death at 50 y/o due to infection
3	35 M	15	SI, spine, hip	NSAIDs, SAZ	8.8/80/60.2	Bloody diarrhea, fever/ anemia	Corticosteroids/mSAZ with relapse, ADA 40 mg every 2 to 4 week	No relapse for 6.7 years after the ADA usage	Alive with lowarthritis activity (BASDAI 2~3)
4	45 F	25	SI, spine, hip, shoulder	MTX, NSAIDs, SAZ	8.1/35/18.8	Bloody diarrhea/anemia	Corticosteroids/SAZ with relapse, ADA 40 mg every 2 to 4 week	No relapse for 5.2 years after the ADA usage	Alive with low arthritis activity (BASDAI 2~3)

ADA: adalimumab, DP: disease period, F: female, IBD: inflammatory bowel disease, M: male, mSAZ: messalazopyrin,

MTX: methotrexate, No.: number, NSAIDs: nonsteroidal anti-inflammatory drugs, SAZ: salazopyrin, SI: sacroiliac,

UC: ulcerative colitis, yr: year

*MTX 10 to 15 mg per week, SAZ 2 to 3 g/ day, NSAIDs replaced with celecoxib after the development of IBD

^†^ESR normal value ≤ 15 mm/hr, CRP normal value ≤ 8 mg/L

^#^High dosages of corticosteroids (1~2 mg/kg/day prednisolone equivalent doses) during active colitis, mSAZ 2 to 3 g/day.

The published English articles related to the efficacy of TNF blockades for the AS-associated IBD manifestation are demonstrated in Table 2 with the enrolled cases dominant in the Caucasian race from Europe and North America, and most studies examine the effect of infliximab (IFX) and ETA with an open-label design^12–28^. Notably, in comparison with the efficacy by using the IBD events (flare and new onset) per 100 patient-years, mAbs other than ADA have a better protection effect than the receptor fusion protein ETA (0.1 versus 2.0 events per 100 patient-years). Furthermore, reactivation or exposure of IBD during the ETA treatment in AS patients has been assumed to be related to its unique structure, TNF neutralizing effect, mode of administration and pharmacokinetic characteristics^29^.

**Table 2:** Efficacy of TNF blockades for the AS-related IBD manifestation in literature

**No**.	**Year**	**Country**	**Patient number**	**TNF blockade**	**Effect on IBD (total flare/new onset events)**	Effect on IBD (events per 100 patient-years*)	**Ref**.
1	2001~2006	Canada, Germany,Netherlands	366	IFX	1 CD	0.2	12–18
2	2002~2006	Belgium, Canada, Finland, Germany, Netherlands, UK, Italy, Spain, USA	724	ETA	8 CD, 6 UC	2.0	12–24
3	2006	France, Germany, Netherlands, USA	295	ADA	1 CD, 2 UC	2.3	25,26
4	2008	Canada, Germany, Netherlands, USA South Korea^#^	278	GLM	0	0	27
5	2014	Netherlands	218	CZP	0	0	28

ADA: adalimumab; CD: Crohn’s disease; CZP: certolizumab pegol; ETA: etanercept; GLM: golimumab; IBD: inflammatory bowel disease; IFX: infliximab; Ref.: reference; UC: ulcerative colitis, UK: United Kingdom; USA: United States of America

^#^74% of Caucasian in this study.

*0.1 events per 100 patient-years in 839 AS patients receiving TNF monoclonal antibodies (IFX, GLM and CZP), and 1.8 events per 100 patient-years in placebo group by pooling 670 AS patients not receiving TNF blockade (reference^13,16,19–22,24,26–28^).

## Discussion

Interestingly, in comparison with a high incidence of IBD in the West with 8 to 14 per 100,000 for UC and 6 to 15 per 100,000 for CD^30^, there is a very low incidence of IBD in Han Chinese with 1.17 per 100,000 for UC and 0.40 per 100,000 for CD, higher in UC than CD^31^. Indeed, in this cohort, there is a lower frequency of histopathology-proven IBD in 0.4% Han Chinese patients with the UC manifestation alone as compared with 5 to 10% of white Caucasian with both UC and CD presentations^2,5^. At the development of colitis, all reported cases had a long-term disease duration, high BASDAI, elevated ESR/CRP levels and advanced joint involvement refractory to the usage of NSAIDs and salazopyrin. Moreover, these patients had the recurrent IBD episodes despite the corticosteroids and messalazopyrin/salazopyrin therapy, leading to the infection-related mortality in 2 cases. Notably, after the prescription of ADA in another 2 patients, starting from 40 mg every 2 weeks and gradually tapered to every 4 weeks, there were no more relapses of colitis and lower arthritis activities with a follow-up period of 6.0 ± 1.1 years, implicating a therapeutic benefit of ADA usage for the AS-related UC manifestation in Han Chinese.

Regarding the efficacy of TNF antagonists for the AS-related IBD manifestation from the literature, the receptor fusion protein ETA appears to have much inferior protection effects as compared with mAbs (Table 2). The American College of Rheumatology strongly recommends the treatment with TNF mAbs over ETA in adults AS patients with IBD^32^. Furthermore, according to the 2016 ASAS-EULAR management recommendations of axial spondyloarthritis for the therapeutic efficacy of different TNF blockades on extra-articular manifestations, mAbs are effective in the treatment of IBD and in preventing the recurrence of uveitis, whereas ETA has shown no efficacy in IBD and contradictory results for uveitis^33^. Notably, in one large-scale, randomized, double-blind, controlled trial in AS patients with more than 95% Caucasian receiving the ADA injection 40 mg biweekly for 24 weeks, 2 cases experienced a UC flare (1.9 events per 100 patient-years versus none in the placebo group) despite a favorable outcome reported from the management of moderately to severely active UC patients under a similar therapeutic schedule^18,26,34^. Nevertheless, in this study, AS patients complicated with UC were successfully treated with the regular ADA injection without relapses under a long-term follow-up. For the usage of ADA in AS-associated UC manifestation, these observations suggest a beneficent effect in Han Chinese in contrast to the unfavorable response in Caucasian. Such a discrepancy in the therapeutic effects implicates a crucial role of ethnic factor in the clinical responses to the biologics therapy^7,35^. Further international collaborations on the large-scale trials are needed to evaluate such an unsettled issue.

Currently, all FDA-approved TNF mAbs have the indication to treat AS or IBD patients^1,6^. In Han Chinese, favorable outcomes from the ADA usage have been observed in rheumatological disorders other than AS like rheumatoid arthritis, psoriasis and psoriatic arthritis^36–38^. For the AS-related uveitis, in addition to no recurrences after the ADA therapy in this study, an earlier report from China demonstrates the clinical response to TNF blockades; however, monotherapy of ETA is not as effective as ADA in preventing the recurrence unless with the additional methotrexate usage^39^. Furthermore, regarding the ADA therapy in CD, the efficacy has been demonstrated in moderate to severe victims in Taiwan with more stringent clinical usage criteria than in western countries, whereas higher remission rates are observed in patients from China than those in clinical trials from the western countries^40^. Interestingly, all cases in this study received regular salazopyrin treatment before the development of IBD, and relapses of colitis occurred despite the daily usage of messalazopyrin, raising an concern regarding the efficacy of salazopyrin/messalazopyrin in protecting or treating the UC manifestation in AS patients with the Han Chinese ethnicity.

In conclusion, in this monocentric Han Chinese cohort, we demonstrate the rarity of AS-associated IBD manifestation with a beneficent effect from the ADA therapy.

## Methods

### Ethics statement

The Institutional Review Board of National Cheng Kung University Hospital (NCKUH) approved this study, and informed consent was obtained from all subjects. All methods relating to humans were performed in accordance with the relevant guidelines and regulations.

### Patient enrollment

This retrospective study was carried out to analyze Han Chinese AS patients with a regular follow-up at the Outpatient Department of NCKUH, a 1,200-bed medical center locating in southern Taiwan, from September 2006 to August 2016.

### Data collection and analysis

The diagnosis of AS was according to the 1984 revised New York diagnostic criteria^41^, not including the juvenile spondyloarthritis. In addition, those patients who had the onset of IBD manifestation before or concurrently with their AS diagnosis were excluded from this study. The radiographs of SI joints were evaluated by two examiners (one radiologist and one rheumatologist) to avoid the observer variation, and computed tomography and/or magnetic resonance imaging were performed in cases with equivocal findings on X-rays. Demographic, clinical, radiological, laboratory and pathological data were analyzed, and their disease activities in spinal involvement were measured with the BASDAI. A detailed review was performed in the medication profiles for biologics, DMARDs, corticosteroids and NSAIDs. Data was expressed as the mean and standard deviation in this study.

### English literatures review

English literature from PubMed was reviewed for the reported IBD manifestation in AS patients with the usage of TNF blockades for their therapeutic outcomes.
